# Lead exposure and the 2010 achievement test scores of children in New York counties

**DOI:** 10.1186/1753-2000-6-4

**Published:** 2012-01-23

**Authors:** Jillian C Strayhorn, Joseph M Strayhorn

**Affiliations:** 1 Undergraduate student, Cornell University College of Arts and Sciences, 147 Goldwin Smith Hall, Ithaca, NY 14853, USA; 2 Department of Psychiatry, Drexel University College of Medicine, Allegheny Campus, 320 East North Avenue, Pittsburgh, PA 15212, USA

**Keywords:** Lead toxicity, lead exposure, achievement, school performance

## Abstract

**Background:**

Lead is toxic to cognitive and behavioral functioning in children even at levels well below those producing physical symptoms. Continuing efforts in the U.S. since about the 1970s to reduce lead exposure in children have dramatically reduced the incidence of elevated blood lead levels (with elevated levels defined by the current U.S. Centers for Disease Control threshold of 10 μg/dl). The current study examines how much lead toxicity continues to impair the academic achievement of children of New York State, using 2010 test data.

**Methods:**

This study relies on three sets of data published for the 57 New York counties outside New York City: school achievement data from the New York State Department of Education, data on incidence of elevated blood lead levels from the New York State Department of Health, and data on income from the U.S. Census Bureau. We studied third grade and eighth grade test scores in English Language Arts and mathematics. Using the county as the unit of analysis, we computed bivariate correlations and regression coefficients, with percent of children achieving at the lowest reported level as the dependent variable and the percent of preschoolers in the county with elevated blood lead levels as the independent variable. Then we repeated those analyses using partial correlations to control for possible confounding effects of family income, and using multiple regressions with income included.

**Results:**

The bivariate correlations between incidence of elevated lead and number of children in the lowest achievement group ranged between 0.38 and 0.47. The partial correlations ranged from 0.29 to 0.40. The regression coefficients, both bivariate and partial (both estimating the increase in percent of children in the lowest achievement group for every percent increase in the children with elevated blood lead levels), ranged from 0.52 to 1.31. All regression coefficients, when rounded to the nearest integer, were approximately 1. Thus, when the percent of children showing elevated lead increases by one percent, the percent of children in the lowest achievement group, according to the regression equations generated, also increases by about one percent. All associations were significant at the 0.05 level.

**Conclusion:**

Despite public health advances, and despite the imprecision of measures, an association between the incidence of elevated blood lead and achievement in New York counties is still apparent, not attributable to confounding by income. Efforts to reduce lead exposure should persist with vigor.

## Background

When researchers in the last part of the twentieth century demonstrated that lead levels in children too low to produce obvious physical symptoms do great harm to their cognition and behavior, they launched a major advance in public health. Clinical symptoms of lead toxicity, including abdominal pain, clumsiness, headaches, gross behavioral changes, and worse, typically become visible in children at blood lead levels greater than or equal to 60 micrograms/deciliter [1]. In the 1970s, the U.S. Centers for Disease Control defined acceptable blood lead level accordingly, using 60 μg/dl as the threshold for concern [1]. In 1979, Needleman et al. [2] demonstrated that dentine lead levels in groups of children without clinical evidence of lead poisoning were significantly associated with measures of cognitive as well as social functioning (including IQ tests, measures of verbal and auditory processing, and school behavior measures). Extensive subsequent research produced similar findings over a wide variety of geographic locations [3-6]. A meta-analysis performed by Needleman and Gastonis in 1990 consolidated the findings of twelve of these subsequent studies into strong evidence for the fact that lead causes significantly impaired cognitive functioning in children at blood levels far below the threshold of concern of the 1970s [7].

Further research on the effects of pediatric lead exposure on IQ specifically has quantified the average IQ loss associated with certain changes in blood lead level. Several studies have estimated a 1 to 5 point decline in IQ for every 10 μg/dl increase in blood lead [8]. Several others have focused on the cognitive effects of lead at the lower end of the lead exposure spectrum: Bellinger, Stiles and Needleman found an average decrease of 5.8 IQ points with a change in blood lead from 0 μg/dl to 25 μg/dl; Schwartz reported an average decrease of 2.6 IQ points with an increase from 10 μg/dl to 20 μg/dl; Lanphear et al. estimated an average decrease of 6.9 IQ points with an increase from 2.5 μg/dl to 30 μg/dl [9-11]. And others have explored the idea that the relationship between blood lead and IQ is non-linear, and that much of the damage is in fact done over the first few micrograms per deciliter. In 2003, for example, Canfield et al. found that an average blood lead increase from 1 μg/dl to 10 μg/dl was associated with 7.4 point decline in IQ [12]. It is still unknown whether there is any level of lead exposure below which harmful effects do not occur; current CDC guidelines set a threshold of 10 micrograms per deciliter. Throughout this article, we will refer to this level of 10 μg/dl as a threshold for concern, and levels above this as "elevated," without implying that a biological mechanism corresponds to this arbitrary cutoff point, and particularly without implying that levels below this are harmless.

The influences of lead exposure on cognitive and social functioning have been noticed with a wide variety of outcome variables. In a long-term follow-up with the participants in his 1979 study, Needleman and colleagues found that childhood lead exposure was associated, eleven years later, with lower class standing, increased absenteeism, lower vocabulary and grammatical reasoning scores, and more self-reported delinquent behavior, as well as a markedly higher risk of high school dropout and reading disability [13]. Another set of children with elevated blood lead levels scored higher on measures of attention, aggression, and delinquency after other covariates were accounted for [14]. In another group of children, prenatal and postnatal lead exposure was associated with parent and self-reports of delinquency [15]. In adulthood, the social effects of lead exposure can manifest themselves in criminal behavior: the Philadelphia cohort of the Collaborative Perinatal Project found that childhood lead exposure was the strongest predictor of adult criminality [8]. Several ecological investigations have found societal lead levels to be associated with levels of violent crime [8]. Thus the effects of lead exposure can be noticed in variables less precisely quantifiable than IQ. We see that lead has the potential to impact individuals' functioning across a wide variety of domains, with the ultimate effect of impairing the functioning of society.

Gradually, the research findings of the late twentieth century were translated into action. In 1978, lead paint was banned from residential use in the U.S. [16]. The U.S. Environmental Protection Agency issued its first regulation of leaded gasoline in 1973, which initiated a phase-down process for the next several years; in 1996, the Clean Air Act was amended with an official ban of the sale of leaded fuel for use in on-road vehicles [17]. In 1986, the Safe Drinking Water Act outlawed the repair or installation of leaded pipes, fixtures, solders, and fluxes [18]. By the early 1990s, the percentage of children with blood lead levels above the threshold of 10 μg/dl had fallen dramatically. Whereas 88.2% of children in the United States had blood levels exceeding that threshold between 1976 and 1980, that figure had fallen to 1.6% by 2002 [19].

This story represents an enormous public health success. Has the mission been successfully accomplished? As a USA Today article declared of lead paint in 2007, "It's banned, but not gone" [20]. According to estimates of the American Academy of Pediatrics in 2005, one in four children lives in housing that contains deteriorating lead paint [16]. According to estimates of the National Center for Healthy Housing, renovation, remodeling, and repainting of older housing stock exposes approximately 1.1 million children in the United States to lead annually [20]. And worldwide, children are still being exposed to lead in even larger numbers, often as a result of industrialization in formerly "underdeveloped" countries [21].

How noticeable are the effects of lead on academic performance? In this study we use recent data on lead exposure and school achievement in counties across New York State to explore the continuing effects of lead upon children.

In addition to the sizeable body of research that has examined the influences of lead exposure on IQ, more recent studies have come to focus more on its effects on school achievement. Some have considered variables related to school performance - class rank, teacher ratings, learning and behavior problems, dropout rates - as outcome variables [13,6,22-24]. Others have included individual achievement tests in addition to individual IQ tests. Bellinger, Stiles, and Needleman, for example, found that lead exposure at 2 years of age predicted academic deficits as measured by the Kaufman Test of Educational Achievement at 10 years of age [9]. Lanphear et al. noticed an inverse relationship between lead exposure and the arithmetic and reading subtests of the Wide Range Achievement Test-Revised [25]. Yule et al. found that scores on tests of reading, spelling, and IQ were negatively associated with blood lead levels [26]. Fulton et al. noticed a dose-response relation between test scores on the British Abilities Scale and lead exposure [3]. And Surkan et al. reported lower scores on the reading and math components on the Weschler Individual Achievement Test among children with even slightly elevated blood lead levels [27].

A study using school achievement test scores, carried out by Miranda et al. [28], assessed the relationship between early childhood lead exposure and school achievement, using the individual child as the unit of analysis. More than 8,000 children were in the sample. Their results indicated a dose-response effect between lead exposure and achievement on end-of-grade tests, for blood lead levels below 10, and even below 5, μg/dl. A subsequent study led by Miranda verified these conclusions with a much larger sample (over 57,000) of North Carolina school children, across all one hundred counties [29]. A third study by Miranda et al. established a dose-response relationship for designations of exceptionality in the North Carolina dataset; the slope of the dose-response curve was even higher in the range between the first 1 and 5 μg/dl than between 5 and 10 [30].

In the current study, we used the results of regular academic testing in New York State, as well as separately published data on lead levels and income, to examine whether the academic performance of children in New York counties is associated with the incidence of elevated blood lead in those counties. Using publically accessible data, reported for the counties of New York, exclusive of New York City, we obtained statistics influenced by thousands of children. We included income data in the hope of controlling for socioeconomic status, addressing the question of how much the incidence of elevated blood lead predicts achievement, above and beyond the prediction accomplished by income.

## Methods

### Sample

The unit of analysis for our study was not the individual child, but the 57 New York counties lying outside New York City. Lead data were not available to us for the counties of New York City.

For the 57 counties outside New York City, the New York State Department of Health publishes the results of mandated blood lead testing. We used these results as estimates of the incidence of elevated blood lead, county-by-county, over two periods: from 2003 to 2005, and from 2005 to 2007. The number of children tested in 2004, 2005, 2006, and 2007 ranged from 193,239 to 207,452 [31,32].

To estimate county-by-county school achievement, we used the 2010 results of the New York State Testing Program for 3^rd ^graders and 8^th ^graders. Among third graders, 196,604 took the English Language Arts (ELA) exam and 198,785 took the mathematics exam [33]. Among 8^th ^graders, 204,383 took the ELA exam and 206,739 took math [34].

### Measures

New York State requires that all children receive blood lead testing at age one and age two, as well as an assessment of the risk of lead exposure at least annually from six months to six years of age; if the assessment indicates reason for concern, health providers are required to readminister tests [35]. The State Department of Health records the number of children newly identified as having elevated blood lead levels (defined as greater than or equal to 10 μg/dl) each year in the form of an incidence rate, the number of new cases per 1,000 children tested [36]. To adjust for variability in rates, the reported state-wide incidence rate and county incidence rates are an average of the rates recorded in each of three years. We used two sets of county incidence rates: the averaged rates from 2003, 2004, and 2005, and the averaged rates from 2005, 2006, and 2007.

Regular state-wide academic testing occurs as a result of New York State requirements, complying with the No Child Left Behind Act, with exams in math and English Language Arts (ELA) administered annually to all students in grades 3 through 8. The test questions are developed by a team of writers who are trained according to the New York State Learning Standards and test specifications; drafted questions are reviewed by CTB/McGraw-Hill, New York State teachers and administrators, and staff of the Department of Education before being field tested. Only the questions found to be reliable and valid are included in the final operational test forms [37]. The assessment results are presented county-by-county in terms of both the mean test score and the percentage of children scoring at one of four levels: Level 4 (Exceeds Proficiency Standard), Level 3 (Meets Proficiency Standard), Level 2 (Meets Basic Standard), and Level 1 (Below Standard) [38]. The standards are defined according to research carried out by New York State Testing Advisory Group and CTB/McGraw-Hill [38]. Our analyses used the percentage of 3^rd ^and 8^th ^grade students scoring Below Standard in math and ELA in 2010 to estimate county academic performance, because this metric intuitively corresponds most closely to the lead data - the lead results are presented as the proportion of children testing over the threshold level. We also used academic test results from 2006 to estimate the stability of the counties' achievement levels over time.

We converted the incidences of elevated lead levels to percents by dividing the published "per thousand" rates by 10. Thus the rates of children over the lead threshold and the rates of children in the lowest achievement group are in the same units, i.e. percents.

To control for socioeconomic status in our analysis, we used published median household incomes for New York State counties, calculated by the Small Area Income and Poverty Estimates program (SAIPE). The program, sponsored by the U.S. Census Bureau and supported by other federal agencies, was created with the goal of providing estimates that are more current than the decennial census, combining "data from administrative records, intercensal population estimates, and the decennial census with direct estimates from the American Community Survey to provide consistent and reliable single-year estimates" [39]. The income data we used in this study represent the SAIPE's estimates of the 2004 median household incomes and the 2008 median household incomes in New York State counties.

### Sources of Data

Each set of county lead levels is available on the New York State Department of Health's website, in the 2004-2005 Surveillance Report Section 2 [40] and the 2006-2007 Surveillance Report Section 2 [41]. The county academic achievement data are available through the Information and Reporting Services of the New York State Department of Education, under English Language Arts and Mathematics Assessment Results [33,34]. The median income data are among the data offered by the Small Area Income and Poverty Estimates program, available on the U.S. Census Bureau website [42].

### Analytic Methods

Our analyses tested the association between incidence of elevated lead and achievement in two cohorts and two subject areas: 3^rd ^graders and 8^th ^graders in 2010, and English Language Arts (ELA) and mathematics achievement test scores. For each association, we first examined the bivariate relationship between lead and achievement. We report unit-free effect size estimates with Pearson correlations, and unit-dependent estimates with bivariate regression coefficients. These regression coefficients answer the question, "For each percent increase in children with elevated lead, by how much does the predicted percent of children in the lowest achievement level increase?"

For 3^rd ^graders, we used the lead levels averaged over 2005-2007; for 8^th ^graders, we used lead levels averaged over 2003-2005. Thus for each cohort, we used as the measure of lead burden the incidence rate in that county for several years before the test occurred.

Next, we checked to see if socioeconomic status is a possible confounding variable. Past research indicates that socioeconomic status is associated with both academic achievement [43] and lead burden [44]; the relationship among the three variables has prompted some scholars to characterize lead as an environmental contributor to the achievement gap [29,30]. To check whether these previously found associations with socioeconomic status hold in our sample, we computed correlations of income with incidence of elevated blood lead and with achievement.

Finally, we re-tested our main hypothesis, statistically removing the effects of family income. For 3^rd ^graders, we used 2005-2007 lead levels and median income data from 2008; for 8^th ^graders, we used 2003-2005 lead levels and median income data from 2004. (We used median income data from a time close to that in which the lead levels were measured.) We calculated partial correlations, unit-free measures of the association between lead and achievement with the effects of income statistically removed from each variable. We then calculated unit-dependent partial regression coefficients, the coefficients for incidence of elevated blood lead in the multiple regression equation in which both incidence and median family income have been entered. These regression coefficients are meant to answer the question, "How much does the incidence of elevated blood lead predict achievement, over and above the prediction accomplished by income?" We calculated confidence intervals for all statistics we report.

## Results

### Descriptive Statistics

The descriptive statistics (mean, standard deviation, minimum and maximum) are reported for each of our eight variables (ELA grade 3, ELA grade 8, Math grade 3, Math grade 8, Lead 2003-2005, Lead 2005-2007, Income 2004, and Income 2008) in Table 1. We examined histograms for each of these variables. The distributions of all variables appeared roughly normal, with the exception of the income variables. The income variables were, unsurprisingly, skewed to the right.

**Table 1:** Descriptive Statistics

Variable	Mean	Standard Deviation	Minimum	Maximum
Percent scoring at lowest level, ELA grade 3	12.2	2.7	7.1	18.8
Percent scoring at lowest level, ELA grade 8	6.9	1.8	3.2	11.1
Percent scoring at lowest level, math grade 3	8.6	2.5	3.3	15.9
Percent scoring at lowest leve, math grade 8	7.5	2.4	3.8	13.3
Percent with blood lead > 10, 2003-2005	1.69	1.07	0.31	5.3
Percent with blood lead > 10, 2005-2007	1.38	0.89	0.20	4.14
Income, 2004	42,726	9,623	33,791	75,514
Income, 2008	51,095	12,948	39,821	94,856

N = 57 counties for all variables.

The sample size for all analyses reported in this article was 57 counties. Within these counties there were no missing data cells for any variable.

### Stability of Measures over Time

The reported average lead levels from 2003-2005 and from 2005-2007 were very highly correlated, with r = 0.91 (p < 0.0005). Median household incomes were as well; the correlation between county income in 2004 and income in 2008 was 0.99 (p < 0.0005). We checked the stability of the academic variables by computing the correlation between the 2006 and 2010 scores for ELA grade 3 and 8 and for math grade 3 and 8. The correlations for those, respectively, were 0.46, 0.59, 0.62, and 0.46 (p < 0.0005 for all). The very high stability of the lead and income variables suggest that recent years' data sets for these variables would be nearly interchangeable for the sake of our analyses. Thus which particular year's lead or income data were used for our analyses probably makes little difference.

### Bivariate Associations Between incidence of Elevated Blood Lead and Achievement

Table 2 presents the bivariate correlations between lead and each of the four achievement variables; these range from 0.38 to 0.47. Table 3 presents the coefficients for lead in the bivariate regression equations where achievement is the dependent variable and lead is the independent variable; these range from 0.73 to 1.31. All of these associations are significantly different from zero at a less than 0.01 level of significance.

**Table 2:** Bivariate Correlation of Lead and Achievement

Academic Outcome	Raw Correlation with Lead Burden	p-value	95% Confidence Interval
ELA 3^rd ^grade	0.38	0.004	(0.13, 0.58)
ELA 8^th ^grade	0.43	0.001	(0.20, 0.62)
Math 3^rd ^grade	0.47	< 0.0005	(0.23, 0.65)
Math 8^th ^grade	0.45	< 0.0005	(0.22, 0.64)

**Table 3:** Bivariate Regression of Lead and Achievement

Academic Outcome	Bivariate Regression Coefficient with Lead Burden	p-value	95% Confidence Interval
ELA 3^rd ^grade	1.15	0.004	(0.39, 1.91) SE = 0.377
ELA 8^th ^grade	0.73	0.001	(0.32, 1.14) Se = 0.204
Math 3^rd ^grade	1.31	< 0.0005	(0.65, 1.97) Se = 0.337
Math 8^th ^grade	1.01	< 0.0005	(0.47, 1.55) Se = 0.268

### The Relationship of Income to incidence of Elevated Blood Lead and Achievement

As expected, lead exposure was negatively related to income level. The correlation between median family income in 2004 and the percent of children with blood lead greater than 10 μg/dl in 2003-2005 was -0.36 (p = 0.005). The correlation between median family income in 2008 and the percent of children with blood lead greater than 10 μg/dl in 2005-2007 was -0.40 (p = 0.002).

Also as expected, income level was related to achievement, although not as highly in our data set as in some others [39]. The correlations between median family income in 2008 and the percent of 3^rd ^graders in the county scoring at the lowest level on the ELA exam and the math exam were -0.33 (p = 0.013) and -0.35 (p = 0.007), respectively. The correlations between median family income in 2004 and the percent of 8^th ^graders scoring at the lowest level on the ELA exam and the math exam were -0.25 (p = 0.058) and -0.46 (p < 0.0005), respectively.

Our results are in accord with previous findings that lead exposure is higher in families of low socioeconomic status [42], and that school achievement is also lower in families of low socioeconomic status [41].

### The Relationship of Lead and Achievement with the Effects of Income Removed

Table 4 presents the partial correlations between lead and achievement, controlling for income; these partial correlations range from 0.29 to 0.40. Table 5 presents the partial regression coefficients for the incidence of elevated blood lead variable in the multiple regression equations in which lead and income are used to predict achievement; these coefficients range from 0.52 to 1.09. All of these associations are significant at a level less than 0.05.

**Table 4:** Partial Correlation of Lead and Achievement, Controlling for Income

Academic Outcome	Partial Correlation Coefficient with Lead Burden	p-value	95% Confidence Interval
ELA 3^rd ^grade	0.29	0.031	(0.027, 0.51)
ELA 8^th ^grade	0.32	0.016	(0.065, 0.54)
Math 3^rd ^grade	0.38	0.004	(0.13, 0.58)
Math 8^th ^grade	0.4	0.002	(0.15, 0.60)

**Table 5:** Multiple Regression of Lead and Achievement, Controlling for Income

Academic Outcome	Partial Regression Coefficient with Lead Burden	p-value	95% Confidence Interval
ELA 3^rd ^grade	0.9	0.031	(0.085, 1.71) Se = 0.406
ELA 8^th ^grade	0.52	0.016	(0.10, 0.93) Se = .207
Math 3^rd ^grade	1.09	0.004	(0.36, 1.81) Se = 0.362
Math 8^th ^grade	0.92	0.002	(0.34, 1.50) Se = .289

## Discussion

Despite the great advances in the control of lead over the past several decades, the effects of county lead burden upon the academic achievement of 3^rd ^and 8^th ^graders in 57 New York State counties are clearly visible in the results of 2010 academic testing. The association between elevated lead and low achievement remains after county median income is statistically controlled for. Of the sixteen 95% confidence intervals we report for the association between lead and achievement, none encompasses a zero effect. For 8^th ^grade math, the prediction of 2010 scores by lead data was approximately as strong as was the prediction by 2006 achievement test scores. The fact that lead predicts achievement results even nearly as strongly as previous achievement results do is remarkable.

Our regression coefficients, both bivariate and partial, range from a minimum of 0.55 to a maximum of 1.31. All are in the vicinity of 1. A coefficient of 1 has an easily understood interpretation: for every percent increase in the children with elevated blood lead, the predicted rate with which children fall into the lowest achievement group also goes up by one percent.

The squares of our correlation coefficients represent the fractions of variation in achievement numbers that are "accounted for" by variations in lead levels. From our bivariate correlations, incidence of elevated blood lead accounts for 14% to 21% of the variation in county achievement levels; from our partial correlations, the percent of the variance in "residual achievement after income is removed" accounted for by "residual incidence of elevated blood lead after income is removed" ranges from about 8% to 16%.

In interpreting our statistics, it is important not to apply our coefficients to the relationship between lead and achievement at the individual level - we are accounting for the variation in rates for counties, not individual achievement scores for children.

The results of other studies reviewed, particularly those of Canfield et al. [12] and Miranda et al.[28-30], suggest strongly that the incidence of blood levels above the CDC threshold is a "tip of the iceberg" type measure - the higher the number of children over the threshold is, the higher the number of children under the threshold but also negatively affected will be.

It is possible that parental education level is a confounding variable that may account for some of the relationship between the incidence of elevated lead and achievement and may not be sufficiently removed by controlling for median family income. If this is the case, the "true" relationship between lead and achievement would be less than our reported correlations because of residual confounding. On the other hand, it is possible that the predominantly correct causal model is that low income causes people to live in substandard housing, which causes elevated lead levels, which cause lower achievement. If this is the case, then removing the effects of income would "partial causes from effects" and yield an underestimate of the association. In this case, the most accurate estimate from our data for the relation between lead and achievement would probably lie closer to the bivariate coefficients than to the partialled coefficients. In any case, the association between lead and achievement does seem to hold up even after partialling the socioeconomic variable that we had access to, which was a reasonable candidate for a confounder.

In observational studies such as this one, it is customary to caution that observed correlations do not imply causation. However, as with cigarette smoking and health outcomes, the total literature on lead and intellectual functioning leaves no doubt that a causal relationship exists.

All the measures used in this study are fairly gross indicators of the underlying phenomena. Less than 100% of children are tested for lead, despite mandates. The metric of percent of children above the CDC threshold discards important information as to exactly how high the lead levels were. We did not know and thus could not take into account the proportions of children who are eliminated from state testing because of disability. No one knows how many children failed to give maximal effort on the group achievement tests. The careful checking of each achievement test that is possible in studies using individual testing is not possible with data gathered on a couple of hundred thousand children. Although it is conceivable that biases in these measures could have elevated the associations that we found, our guess is that the error variance would be more likely to attenuate the relations between the incidence of elevated blood lead and achievement. We interpret the fact that the association is clearly visible despite these sources of error as a testimony that a signal exists within the noise.

If it is true that "the remaining major source of lead [in the United States] is older housing stock," [1] our results do tend to strengthen the case of those who advocate removing lead from existing housing. According to a study carried out by Grosse et al., the average decline in blood lead levels in children age 1-5 from 1976 to 1999 was 15.1 μg/dl, which resulted in an estimated 2.2-4.7 increase in IQ points [45]. From this, they estimated the IQ-related increase in worker productivity and income, concluding that the economic benefits for each year's cohort of 3.8 million children 2 year-olds range from $110 billion to $319 billion. Needleman summarized such results in 2004: "Although the cost of lead paint abatement is measured in billions of dollars, the monetized benefits of such a Herculean task have been shown to far outweigh the costs" [1].

In the U.S., despite the great emphasis and attention given to achievement test scores, and despite evidence indicating that those scores could be increased by lead abatement, the political will to carry out and enforce such procedures appears to be declining. Major candidates for president of the U.S. have taken strong stands against even the existence of the Environmental Protection Agency [46].

Achievement test scores are not important in and of themselves, but the human potential that these scores imperfectly reveal is of huge importance. It is safe to estimate that millions of children are still having their potential reduced by lead. The continuing effects of this known neurotoxin are so large that our analyses, which used data gathered and published for reasons other than our investigation, demonstrate that the ill effects are ongoing.

## Conclusion

Despite the dramatic progress that has been made in the reduction of lead exposure in the United States, it remains true that a significant portion of the variance in achievement levels in New York counties can be accounted for by the incidence of elevated blood lead levels. While this study indicates only association and not causation, the known neurotoxic effects of lead make it very plausible that there is a causal relationship between lead exposure and decreased achievement. Vigorous efforts should be made to decrease children's exposure to lead; complacency should not result from the improvements that have been made to date.

## Competing interests

The authors declare that they have no competing interests.

## Authors' contributions

JCS reviewed the literature, constructed the tables, wrote the manuscript, and compiled, checked, and wrote the reference information. JMS conceived the study, found the data sets, did a preliminary analysis, directed the subsequent analyses, and edited the manuscript. Both authors working together entered the data, checked data entry, and carried out the statistical analyses. Both authors read and approved the final manuscript.
